# MyDispense simulation in pharmacy education: a scoping review

**DOI:** 10.1186/s40545-023-00618-0

**Published:** 2023-09-28

**Authors:** Harjit Kaur Khera, Emily Mannix, Reem Moussa, Vivienne Mak

**Affiliations:** Faculty of Pharmacy and Pharmaceutical Sciences, Monash University, Parkville, VIC Australia

## Abstract

**Background:**

MyDispense is a free online virtual simulation software developed by Monash University. The software facilitates students to practise, apply and hone the skills of a pharmacist in a realistic environment without the potentially life-threatening consequences of a real-life error. Although the focus of MyDispense was initially on exposure to community pharmacy practice scenarios, its modular build and customizability, indicate that there are a wide range of applications that could be incorporated into pharmacy education. Therefore, this study reviews and appraises the literature on the use of MyDispense within pharmacy education.

**Methods:**

A scoping review was conducted. The electronic databases (CINAHL, Ovid Embase, Ovid Medline, Google Scholar, and Scopus) were searched to identify scholarly articles related to MyDispense in pharmacy education from January 2011 and August 2022.

**Results:**

Forty-three papers met the inclusion criteria and were analysed in this scoping review. A total of 418 key sentences and segments of text were extracted from the papers and subsequently categorized into 10 subthemes. The 10 subthemes were dispensing skills, communication skills, decision-making/problem-solving skills, student performance, pharmacy law, applying theoretical knowledge, support educators, pharmacy practice, feedback/reflection and practice in a risk-free environment. In total, four overarching themes summarize how MyDispense is supporting pharmacy education: supporting education; skill development; application of knowledge and student outcomes.

**Conclusion:**

The scoping review found that MyDispense was mostly used to support education and student skill development. These findings can be used to support pharmacy educators globally on the various uses and applications of MyDispense in their teaching.

**Supplementary Information:**

The online version contains supplementary material available at 10.1186/s40545-023-00618-0.

## Background

The integration of computer-based simulation within pharmacy education has been a valuable tool for both students as well as pharmacy educators [1]. There’s been improvement in students’ performance in clinical activities [1] as well as in knowledge retention and transfer [1]. On the other hand, pharmacy educators found that simulation filled a need for faculty and clinical site resources as the simulation could provide structured and prompt feedback to students [2]. However, the challenge faced by many pharmacy education programs globally are the overhead costs associated with these computer-based simulations including set-up, maintenance, staff training, and technical support [2]. As such, these factors have staggered the widespread use of these valuable resources within pharmacy education [3].

MyDispense is a free online virtual simulation software developed by and introduced into the Faculty of Pharmacy and Pharmaceutical Sciences at Monash University, specifically for pharmacy education in 2011 [4]. The software facilitates students to practise, apply and hone the skills of a pharmacist, from beginner to highly advanced, in a realistic environment without the potentially life-threatening consequences of a real-life error [5]. In particular, it allows students to experience various case-based pharmacy scenarios, built on simulations of realistic pharmacist–patient interactions from the greeting patients, to filling prescriptions, helping patients with self-care needs, validating the work of virtual colleagues to ensure that medications are accurate, legal, and safe before dispensing [5], as well as the retrieval of medications from shelves/fridges/safes, the provision of medicines and appropriate counseling [6].

MyDispense has encouraged international collaborations between users from over 200 pharmacy schools globally [5]. Accordingly, to enhance international usability and functionality, the software has since been updated and customized in conjunction with user to build country-appropriate versions [5, 7]. Internal evaluations by some of these global partners have praised the simulator’s value within pharmacy education especially in an environment where opportunities to partake in learning opportunities may be restricted [8]. For example, MyDispense was an important tool that allowed educators to deliver teaching and meet student learning outcomes during the Coronavirus pandemic 2019 (COVID-19) when government enforced lockdowns led to a global need for remote teaching strategies [5]. Additionally, the introduction of MyDispense within the pharmacy curriculum at Nelson Mandela University, South Africa, has shown to be beneficial to students who reported that the simulated scenarios encouraged and assisted them in applying their clinical knowledge and to make the right clinical decisions when dispensing medications [9]. This demonstrates that MyDispense is a valuable educational tool that prepares students for workplace pharmacy experiences [10] as the integration of MyDispense exercises into pharmacy curricula improves the delivery of education material to students as well as the application of material to the real-world, thereby providing students with a holistic view of a variety of pharmacy settings [6].

Although the focus of MyDispense was initially on exposure to community pharmacy practice scenarios [6], its modular build and customizability [5] indicate that there are a wide range of applications that could be incorporated into pharmacy education. While systematic reviews on various pharmacy simulation programs also encompassing MyDispense have been reported within the literature, a gap pertaining to the diverse applications and uses of MyDispense within pharmacy education exists. Therefore, the objective of this scoping review is to appraise the current applications of MyDispense within pharmacy education to inform practice, utilization and also advance future development of the software.

## Methods

A scoping review was conducted and the primary objective of this study was to gain a better understanding of the various applications of MyDispense. Scoping reviews are often used to summarize findings from available literature on topics that have not been previously widely reviewed. To ensure reliability and reproducibility of the method, the scoping review was based on the Arksey and O’Malley framework [11].

### Search strategy

A systematic search strategy was used as part of this review. The electronic databases (CINAHL, Ovid Embase, Ovid Medline, Google Scholar, and Scopus) were searched to identify scholarly articles related to MyDispense in pharmacy education. Other references were also identified by examining the bibliographies of papers that met the eligibility criteria, and via searching by hand. The database searches involved two stages using a combination of keywords, Medical Subject Headings (MeSH), and/or CINAHL subject headings (Table 1). In the first stage, the search strings focused on MyDispense, virtual simulation, and other pharmacy-related simulations. The second stage focused on pharmacy education. The outcomes of both search strategies were collectively evaluated.

**Table 1:** Review search terms and databases reviewed

Databases	Search strategy*		
	Concept 1		Concept 2
Ovid Medline and Ovid Embase	MyDispense (map to subject heading, select “Computer Simulation” and “Simulation Training”) OR “MyDispense” (not mapped to subject heading) OR “Simulation ADJ2 (education OR learning OR virtual OR patient)”	AND	Pharmacy (map to subject headings, choose subheadings: “Community pharmacy services”; “Education, pharmacy”; “Education, Pharmacy”, “Continuing; Education, Pharmacy”, “Graduate; Pharmacy”; “Pharmacy Residencies”; “Students, Pharmacy”; “Faculty, Pharmacy”; “Schools, Pharmacy”)” OR “Pharmacy ADJ2 (curriculum OR placement OR education OR university OR degree OR undergraduate)
Scopus	(Simulation W/2 (education OR learning OR virtual OR patient)) OR MyDispense)		Pharmacy W/2 (curriculum OR placement OR education OR university OR degree OR undergraduate)
CINAHL	(Simulation NEAR/2 (education OR learning OR virtual OR patient)) OR MyDispense)		“Pharmacy NEAR/2 (curriculum OR placement OR education OR university OR degree OR undergraduate)”
Google Scholar	MyDispense		Pharmacy

*Limited to January 2011- August 2022

### Eligibility criteria

Papers were included in the scoping review if they were: (1) published sources; (2) peer-reviewed; (3) contained text that referred to the utilization of MyDispense within pharmacy education; (4) published from January 2011 and August 2022. The start date for the search is aligned with the launch on MyDispense in 2011 [3]. Papers were excluded if they were: (1) published in a language other than English and (2) MyDispense use in pharmacy education was not the major focus of the paper.

### Study selection and extraction

Retrieved articles were stored and shared with the other researchers using the Endnote digital referencing software. A template used for extracting data and reviewing papers was developed and agreed by all team members. Five team members were involved in the data extraction. The data extracted included: (1) titles of paper; (2) author’s name; (3) year of paper; (4) journal; (5) country of study; (6) duration of study; (7) aims/objectives; (8) methods/study design; (9) summary of results; (10) outcomes; (11) extracts; (12) code; (13) subthemes and overarching themes and (14) keywords. The definitions for extracts, code, subthemes and overarching themes are given in Table 2.

**Table 2:** Definitions used during data collection

Heading	Definition (if applicable)
Extracts	Referred to key sentences and segments of text from the paper
Code	Terms used to generate meaning and referred to the label assigned to the extracts
Subthemes and overarching themes	Refers to grouping of codes, a subtheme referred to a category and a theme is an overarching group of categories that related back to the initial research question [12]

The included papers were read, and key quotes and results collected as *extracts* with recurrent patterns identified via thematic analysis. These were then discussed amongst the team to decide and agree upon different *codes* that summarized the meaning and critical points of each *extract*. Any differences between team members’ interpretations were resolved through discussion and consensus. The *themes* are derived from the analysis and is an overarching subject of a group of categories and related to the initial research question. The *subtheme* is derived from a grouping of *codes* forming a category.

Once data were collated in the template, all five team members met to discuss the subthemes and overarching themes and that emerged [12]. Any disagreements were resolved through a discussion and consensus to be reached between team members. When there was persistent disagreement, a senior team member (HS) participated in discussion to achieve consensus.

## Results

**Fig. 1:**
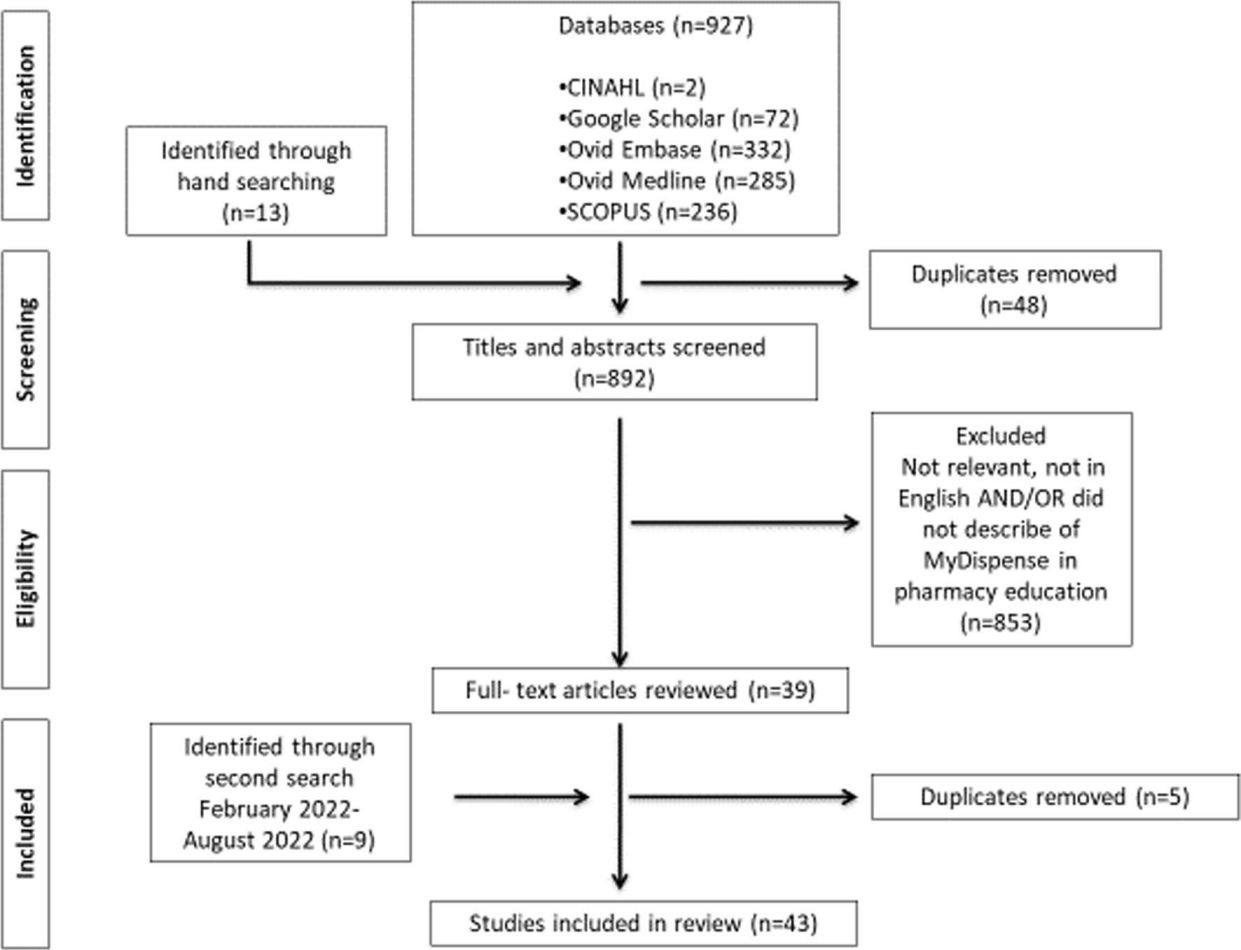
PRISMA flowchart of the literature search and study selection

Figure 1 shows the study selection process. The initial database searches captured 940 papers (CINAHL: 2, Ovid Embase: 332, Google Scholar: 72, Ovid Medline: 285, and Scopus: 236), of which 48 were duplicates. The second search of the databases from February 2022 to August 2022 captured 9 papers (CINAHL: 0, Ovid Embase: 3, Ovid Medline: 1, Google Scholar: 1 and Scopus: 1). Following removal of the duplicates and the application of the inclusion and exclusion criteria, 43 papers met the eligibility criteria and were included in the final review (Additional file 1: Appendix 1).

### Characteristics of included studies

The 43 papers varied in their methodology and in their outcomes; 16 papers used mixed methodology [6, 8, 13–26], 10 were quantitative papers [27–36] and 13 were qualitative papers [5, 7, 9, 10, 37–45]. The majority of papers were conducted in the United States (*n* = 22) [6, 13, 16–19, 21–23, 25, 27–29, 32–35, 39, 42, 44, 46, 47] and Australia (*n* = 7) [7, 15, 36, 37, 43, 45, 48]. Papers involving MyDispense were also conducted in Saudi Arabia (*n* = 2) [8, 49], South Africa (*n* = 3) [9, 10, 26], Malaysia (*n* = 1) [39], Turkey (*n* = 1) [31], Sri Lanka (*n* = 1) [20] and the United Kingdom (*n* = 1) [24]. Five papers report on multi-continent studies, four paper report on a study conducted in the United States and Australia [4, 14, 30, 41], while the other paper reports on a MyDispense study in Saudi Arabia and Australia [40]. The most commonly reported data collection method was surveys (*n* = 28), which were completed by pharmacy student participants to evaluate their perception towards MyDispense following the completion of simulation exercises. Other data collection methods reported within the included literature were focus group (*n* = 1) and observations (*n* = 8).

### Themes and subthemes

A total of 425 extracts were categorized and encompassed within ten subthemes. These subthemes included dispensing skills, communication skills, decision-making/problem-solving skills, student performance, pharmacy law, applying theoretical knowledge, support educators, pharmacy practice, feedback/reflection and practise in a risk-free environment. Four overarching themes that summarize how MyDispense is supporting pharmacy education emerged from these subthemes; these include skill development, student outcomes, application of knowledge and supporting education, these are presented alongside their corresponding subthemes in Table 2 and are italicized in the text.

### Theme 1: skill development

The majority of papers (*n* = 33) [5–10, 13–22, 24, 27, 28, 30–34, 36, 38, 39, 41, 45, 47–49] included in this scoping review mentioned using MyDispense for the purpose of student skill development (Table 2).

#### Subtheme: dispensing skills

This encompassed the use of MyDispense to develop dispensing skills (*n* = 33) [5–10, 13–22, 24, 27, 28, 30–34, 36, 38, 39, 41, 42, 45, 47–49] by practising the many technical aspects such as confirming patient details (*n* = 4) [6, 10, 21, 30], prescription validation and legality checks (*n* = 9)[5, 9, 10, 13–15, 21, 24, 30], reviewing the prescribed medicines for drug interaction (*n* = 2) [14, 22], label preparation (*n* = 9) [6, 10, 15, 17, 21, 22, 45, 48, 49]and product selection (*n* = 6). The medication dispensing process may at times also involve consultation of evidence-based sources for the purposes of clarification, this was mentioned in four papers, whereby students utilized drug resources or databases to guide and improve their dispensing skills (*n* = 4) [13, 21, 32, 38].

#### Subtheme: communication skills

A total of 17 references [7–10, 13, 14, 16–18, 21, 22, 27, 28, 30, 32, 38, 41] mentioned that MyDispense was also used to develop student’s communication skills (Table 2). Specifically, MyDispense was used to facilitate preparation of counselling points allowing students to practise and improve their knowledge on prescription medications, over the counter (OTC) products and self-care (*n* = 10) [7, 9, 13, 14, 16, 18, 21, 22, 32, 41]. The papers also showed that students were also able to develop a comprehensive fact-finding framework for interviewing patients and gathering information which enabled them to practise their communication with other healthcare professionals by asking questions or verifying information (*n* = 6) [7, 9, 14, 21, 22, 30]. Development of student's communication skills in preparation for community placement has also been possible through prior exposure to and practice of typical pharmacy scenarios in MyDispense, whereby students who completed the MyDispense activities were well equipped to provide medication counselling compared to students who did not complete the exercises [18]. In addition, completion of MyDispense scenarios before placements was also associated with students having more engaging patient care interactions while on placement [16]. Although many papers acknowledged the value of MyDispense in developing student’s communication skills, one paper indicated that virtual simulation training cannot entirely replace face-to-face training and it may be detrimental to the learner if their training is solely online [8].

#### Subtheme: decision-making/problem-solving skills

Exposure to scenarios modelled on real pharmacy problems [17] is feasible using MyDispense. This facilitates students to develop decision-making and problem-solving skills (Table 2), mentioned within 4 papers [6, 9, 10, 21], it involves the integration of cognitive processes when performing the technical aspects of dispensing. Another aspect of MyDispense that contributes to the development of these skills is the capacity to intentionally design scenarios to include various tasks and interruptions requiring students to prioritize and manage their workflow [10]. The management of clinical and therapeutic cases of varying complexity by students are also aspects that contribute to the development of this skill (*n* = 4) [6, 9, 10, 21].

### Theme 2: student outcomes

#### Subtheme: student performance

Several papers (*n* = 29) [5, 6, 8, 9, 13–19, 22, 24, 25, 27–31, 33, 35–39, 41, 44, 47, 48] within this review mentioned student performance as an outcome of MyDispense exposure during pharmacy education (Table 2). Most of these papers investigated the confidence of students in performing pharmacist activities such as dispensing prescriptions, counselling patients, solving medication-related problems, or other professional activities [8, 13, 16–18, 25, 27, 28, 31, 36, 44]. Alongside student confidence, MyDispense use also demonstrated improved student grades and average assessment scores [5, 17, 23, 31, 33, 44, 46]. Other measures of student performance following MyDispense utilization included the development student competency in the professional practice setting (*n* = 8) [5, 6, 14, 31, 32, 35, 36, 38] understanding and identification of prescription errors (*n* = 2) [15, 41], achievement of learning objectives [16], and establishing professional values and responsibilities [6].

### Theme 3: application of knowledge

The value of MyDispense is that it can be utilized for skill development as well as the application of knowledge.

#### Subtheme: pharmacy law

More specifically it can be used to teach students to apply pharmacy laws (Table 2). A total of 11 papers described the importance and multifaceted benefits of using MyDispense for legal practice in pharmacy [5, 9, 17, 18, 22, 23, 30, 32, 33, 37, 41]. Amongst the papers, MyDispense was recognized as an effective tool for teaching pharmacy law, especially given that it can be a challenging topic for students [17, 32, 41]. Exercises within MyDispense helped students review pharmacy laws, legal requirements and issues that can typically occur while practising [23, 32, 37] and dispensing prescriptions [21, 22, 32, 33]. It was also used to expose students to and understand laws focusing on controlled prescriptions and dispensing them [30, 37].

#### Subtheme: applying theoretical knowledge

There were 20 papers [5, 6, 8–10, 14–17, 27–30, 32–34, 38, 39, 42, 46] that specifically referred to the use of MyDispense to apply theoretical knowledge (Table 2). Most of these papers (*n* = 7) discussed the use of MyDispense by students in order to apply the learned theory into practice [5, 6, 9, 10, 13, 15, 17]. Within the literature there was also some discussion about the use of MyDispense in the application of medication theory by students (*n* = 14) which included familiarization with prescription-only and OTC medications, their indications, dosage, and counselling points [5, 8, 9, 13, 14, 16, 28–30, 32, 33, 38, 42, 46].

### Theme 3: supports education

Within the literature the application of MyDispense was recognized to support education.

#### Subtheme: support educators

Sixteen papers [5, 7, 8, 10, 19, 21, 26, 28, 32, 33, 36, 40, 42–44, 49] specified that it supports educators in their teaching, particularly during the COVID-19 pandemic in which there was limited opportunity for face-to-face classes. For some educators, MyDispense replaced real-life training and sustained completion of educational outcomes without disruption [8, 40]. Five papers also mentioned that MyDispense was an efficient way for educators to teach practice skills to students [19, 21, 26, 43, 44]. The application of MyDispense to track accuracy and the completion of exercises was mentioned in one paper, this is beneficial to educators as it assesses student performance and can be used to improve student learning strategies [36]. Lastly, collaboration amongst educators can facilitate improved student learning through sharing of ideas and MyDispense cases and this was discussed in two papers [5, 26].

#### Subtheme: pharmacy practice

A total of 29 papers [5, 6, 8–10, 13–15, 17–19, 21, 22, 26–28, 31, 32, 34, 38–44, 44, 46–49] described that MyDispense provided an environment which enabled students to experience typical pharmacy practice (Table 2) including face-to-face training, community placements and introductory pharmacy practice experiences (IPPE). Two papers highlighted the potential of MyDispense to prepare students for future pharmacy practice [26, 46].

#### Subtheme: feedback or reflection

There were 14 papers [5, 7, 8, 15, 17–19, 22, 31, 32, 40, 41, 47, 49] that mentioned that MyDispense allows students to receive prompt feedback/reflection on their work (Table 2). This feedback was shown to improve students' learning, knowledge and performance as it facilitates reflection on performance and appraisal of errors in a safe learning environment [5, 17]. The MyDispense feedback was shown to enhance students' understanding of pharmacy laws, regulations and controlled prescriptions [23]. These qualities of MyDispense also make it a useful tool for educators, increasing efficiency and timeliness of providing feedback to students [5].

#### Subtheme: practise in a risk-free environment

Thematic analysis revealed that there were 17 papers [5, 6, 8, 10, 15–21, 27, 28, 32, 36, 39, 49] that mentioned MyDispense facilitated students to practise in a risk-free environment (Table 2) by providing a safe learning space which mimicked community pharmacy experiences. There were 13 papers [5, 6, 8, 10, 16–21, 27, 36, 39] which noted that MyDispense gave students the ability to practise various skills such as dispensing without causing harm or having real-life consequences to the patient. This offered students the opportunity to repeat MyDispense exercises multiple times, the ability make errors in the dispensing and prescription validation process without causing untoward adverse effects on a real patient, and was discussed in three papers [8, 15, 19]. This is an aspect that students have found helpful [27, 28]; it also enhanced their confidence and competency levels in dispensing, clinical practice and outpatient pharmacy practice [18, 32, 36] as well as their knowledge [6]. Some papers also indicated that the risk-free environment equipped students with the required skills to ensure their successful transition from university to placements or clinical settings [6, 8] and to easily identify medication and prescription errors in their future practice [15].

## Discussion

This review identified various applications of MyDispense and its prevalence in pharmacy education which were categorized amongst four overarching themes: “skill development”; “student outcomes”; “application of knowledge” and “supports education”.

It was found that many papers referred to more than one application of MyDispense (Table 3), the utilization of which was quite prominent during the COVID-19 pandemic. During this time MyDispense posed as a supplementary tool, replacing on-site clinical placements and face-to-face classes, as it alleviated barriers to learning and skill development by empowering students to practise and reinforce their dispensing skills, communication skills and decision-making/problem-solving skills without the risk of causing patient harm [6, 17]. The development of such skills is a core focus of pharmacy institutions to prepare students for their future practice [50]. Previously, methods such as curriculum modification and reflective practice were implemented to assist students with this process [51], however, implementation of simulation programs, such as MyDispense, have become more popular due to their convenience and applicability, this is because although the dispensing process may seem simple, it is time consuming and can be influenced by several factors, including the pharmacist's education, knowledge, professional remuneration, competency and communication skills [52]. Hence, it requires regular practice, and MyDispense was shown to be an effective tool in helping students develop these key skills. However, it should be noted that although MyDispense activities were used to replace in person classes and some learning during experiential placements [53], as acknowledged by a single paper in this review [8], the software does not entirely replace the skills learned face-to-face training and from the spontaneity of direct human interactions [54], yet is an effective tool that can be used in adjunct to prepare graduates for practice.

**Table 3:** Overview of papers included in the scoping study demonstrating the application of MyDispense in pharmacy education

Title of paper	Overarching Theme	Skill development			Student outcomes	Application of knowledge		Supports education			
	Subtheme	Dispensing skills	Communication skills	Decision-making/problem-solving skills	Student performance	Pharmacy law	Applying theoretical knowledge	Support educators	Pharmacy practice	Feedback/ reflection	Practise in a risk-free environment
1. Evaluation of Virtual Dispensing Software to Prepare Students for Introductory Community Pharmacy Practice Experience [13]		X	X		X				X		
2. Implementation of Community Pharmacy Dispensing Software (MyDispense) in an Advanced Pharmacy Practice Course (poster) [14]		X	X		X		X		X		
3. Implementation of Community Pharmacy Dispensing Software (MyDispense) in an Advanced Pharmacy Practice Course (abstract) [27]		X	X		X		X		X		X
4. The Use of Simulation to Improve IPPE-1 Performance [28]		X	X		X		X	X	X	X	X
5. Assessing Student Performance in the Medication Use Process Using Community Pharmacy Simulation (MyDispense) [29]					X		X				
6. A Novel Approach to Pharmacy Practice Law Instruction [37]					X	X				X	
7. A Simulated Learning Environment for Teaching Medicine Dispensing Skills [6]		X		X	X		X		X		X
8. Analysis of Dispensing Errors Made by First-Year Pharmacy Students in a Virtual Dispensing Assessment [15]		X			X		X		X	X	X
9. Analysis of Student Performance Outcomes Using Virtual Dispensing Exercises [30]		X	X		X	X	X				
10. Experiential learning in community pharmacy: Online and remote teaching experience in Malaysian higher education remote teaching experience in Malaysian higher education [38]		X	X		X		X		X		
11. Impact of virtual simulation in self-care therapeutics course on introductory pharmacy practice experience self-care encounters [16]		X	X		X		X			X	X
12. Implementation of a virtual dispensing simulator to support US pharmacy education [39]		X			X		X		X		X
13. Integration of a Community Pharmacy Simulation Program into a Therapeutics Course [17]		X	X	X	X	X	X			X	X
14. Integration of a Virtual pharmacy Dispensing Simulator ''MyDispense'' in an Experiential Education Program to Prepare Students for Community Introductory Pharmacy Practice Experience [18]		X	X		X	X			X	X	X
15. Integration of a virtual pharmacy simulation program “MyDispense” in clinical pharmacy education [31]		X		X	X				X	X	
16. International deployment of a virtual dispensing simulator supporting pharmacy education [19]		X			X			X	X	X	X
17. Learners' Perceptions on Virtual Simulation Using MyDispense in the Philippines [20]		X									X
18. MyDispense impact in compensating summer field training course during COVID-19 pandemic [8]		X	X		X		X	X	X	X	X
19. MyDispense: Lessons from Global Collaboration in Developing a Pharmacy Educational Simulation Tool [7]		X	X	X				X	X	X	
20. MyDispense: Taking pharmacy education into the future together [10]		X	X	X			X	X	X		X
21. Use of MyDispense Among Pharmacy Programs Across the United States [32]		X	X			X	X	X	X	X	X
22. Virtual simulation to personalize student learning in a required pharmacy course [21]		X	X	X				X	X		X
23. What now and what next? The new era of OSCE [40]								X	X	X	
24. Educational Methods and Technological Innovations for Introductory Experiential Learning Given the Contact-Related Limitations Imposed by the SARS-CoV2/COVID-19 Pandemic [22]		X	X	X	X	X			X	X	
25. Meeting pharmacy educational outcomes through effective use of the virtual simulation MyDispense [5]		X			X	X	X	X	X	X	X
26. Simulated learning: Integrating clinical knowledge into the dispensing process [9]		X	X	X	X	X	X		X	X	
27. Using MyDispense to simulate validation of controlled substance prescriptions in a pharmacy law course [33]		X			X	X	X	X	X		
28. Using Technology to Enhance Teaching and Learning in Pharmacy Education [41]		X	X		X	X			X	X	
29. Effects of virtual simulation on students' ability to assess self-care patient cases [46]							X		X		
30. Integration of MyDispense in a Doctor of Pharmacy curriculum in the U.S.: Lessons learned [42]		X					X	X			
31. Connecting two pieces of separate puzzles: A MyDispense experience [43]								X			
32. Integration of MyDispense in an experiential education program to improve student preparedness of prescription processing and medication safety [44]					X			X	X		
33. Use of MyDispense pharmacy simulation program in integrated review of pharmacy law [23]						X					
34. Implementation of a virtual dispensing system (MyDispense) into the M.Pharm. curriculum at the University of Manchester [24]		X			X						
Use of online 35. simulation in a required self-care therapeutics course [25]					X						
36. Use of MyDispense to dispense extemporaneously—prepared formulations [45]		X									
37. Collaborative development of a virtual Pharmacy Practice skills laboratory at the University of Zimbabwe School of Pharmacy [26]								X	X		
38. Student pharmacist performance on an Objective Structured Clinical Examination (OSCE) using community pharmacy simulation (MyDispense) [35]					X						
39. Use of a virtual pharmacy simulation (MyDispense) for teaching dispensing skills in first-year pharmacy students [36]		X			X			X			X
40. Effects of virtual simulation on student pharmacists' ability to assess self-care patient cases [34]		X					X		X		
41. An Introductory Over-The-Counter Simulation For First-Year Pharmacy Students Using A Virtual Pharmacy [47]		X			X				X	X	
42. Incorporation of MyDispense, a Virtual Pharmacy Simulation, into Extemporaneous Formulation Laboratories [48]		X			X				X		
43. A comparison between student performances on objective structured clinical examination and virtual simulation [49]		X						X	X	X	X

Within this scoping review the development of various pharmacist skills was measured as enhanced student self-reported confidence in surveys [16], successful completion of exercises [29] and satisfactory preceptor reports of student performance in IPPE (introductory pharmacy practice experiences) [16] and OSCEs [35]. These evaluations are possible as MyDispense integrates computer-based simulation, virtual patients and formative and summative assessment strategies for educators as well as prompt feedback for students [5]; a feature that not all simulation tools within pharmacy education possess [55].

Although MyDispense was initially designed for the purpose of developing student’s dispensing skills; a fundamental competency for pharmacists [6]. This review indicated that MyDispense is also used for the application of knowledge such as teaching students about and to practise scenarios exemplifying pharmacy law. Pharmacy law underpins pharmacy practice and is fundamental to ensure that a pharmacist practises professionally, legally, ethically and with integrity [56]. Therefore, developing a comprehensive understanding of pharmacy legislation and the competencies required to practise within the laws is crucial towards a pharmacy students’ preparation for future practice. At present, MyDispense emphasizes the legal requirements to be considered when validating prescriptions as well the dispensing process for medicines including controlled substances [30, 33, 37]. Students who used MyDispense for this purpose mentioned that it enhanced their learning and helped them recall pharmacy laws and focus on topics that were challenging [30, 32]. Despite these gains there is limited application of MyDispense in this area. Therefore, the development of additional pharmacy law scenarios combined with the outcome of this review may promote utilization and student learning.

The findings of the review indicate that MyDispense offers considerable support to educators including the ability to track student’s completion and accuracy of exercises [36], student performance [13, 29, 30], their perceptions [22, 25, 32, 47] as well as the ability to provide prompt and consistent feedback [17, 18, 28, 31, 49]. These are useful tools and as they can assist in the improvement of and redesign of teaching tools in order to improve student performance and satisfaction [57]. In addition, the provision of timely feedback to students can improve student learner cognitive skills and knowledge by activating metacognition; the awareness and control of cognition through planning, monitoring, and regulating cognitive activities [58].

It was evident that not all the features are being utilized analogously, rather many continue to utilize if for the sole purpose of practising dispensing skills and assessing students. This may be attributed to the lack of awareness and research about the effectiveness of each application. However, it is expected that the findings would be a valuable tool for MyDispense users and non-user institutions as it will facilitate and encourage application of the education tool and also provide scope for future software development.

There are some limitations to this review. Firstly, the generation of themes for this study was reached through consensus amongst team members and may have been influenced by researcher biases. However, adherence to a standardized methodology and confirming thematic validation by consensus decision-making overcame this barrier. Secondly, a quality assessment was not performed on papers included in this review, given the relative paucity of the data, it was important to include all the research found and hence this was deemed unnecessary.

## Conclusion

This review found that Mydispense is used to enhance student learning, increase academic and practical knowledge, develop essential skills needed to become a pharmacist and to support educators in their teaching. This dispensing simulation was readily adopted by educators during the COVID-19 pandemic when face-to-face practical training was not feasible. This exemplified how the integration of MyDispense into pharmacy curriculums offers new approaches to teaching and learning. The outcomes of this research paves the way for the incorporation of MyDispense into Pharmacy curricula globally, and may also be useful for educators and software developers to consider when expanding the MyDispense program in the future.

### Supplementary Information

**Additional file 1: Appendix 1.** Description of articles that were included in the thematic analysis.

## Data Availability

All data generated or analysed during this study are included in this published article [and its supplementary information files].
