# Supplementary material for: MyDispense simulation in pharmacy education: a scoping review
Source: J Pharm Policy Pract. 2023 Sep 28;16:110. doi: 10.1186/s40545-023-00618-0 (PMC10540382; doi:10.1186/s40545-023-00618-0)
Supplement: Supplementary file 1 — Additional file 1: Appendix 1. Description of articles that were included in the thematic analysis. [file 40545_2023_618_MOESM1_ESM.docx]

**Appendix 1. Description of articles that were included in the thematic analysis**

| Title (author, year) | Country/ies | Study design (Qualitative/ Quantitative/ Mixed study) | Data collection method | Aims/objectives | Results |
| --- | --- | --- | --- | --- | --- |
| Evaluation of Virtual Dispensing Software to Prepare Students for Introductory Community Pharmacy Practice Experience  (Diem Thai and Mohammed A. Islam, 2016) | United States | Mixed | Survey | To determine the impact of using MyDispense on the preparation of students for the Introductory Pharmacy Practice Experience (IPPE). | The simulation is helpful in developing dispensing skills, counselling skills, applying theoretical knowledge, and preparing them for future practice. |
| Implementation of Community Pharmacy Dispensing Software (MyDispense) in an Advanced Pharmacy Practice Course  (Kebodeaux and Sewell, 2016) | United States; Australia | Mixed | Survey | To assess the effectiveness and applicability of My Dispense. | Previous community experience did not affect students' perception of MyDispense. Most students agreed that MyDispense is straightforward to learn, more realistic addressing patient issues exercises compared to papers, learn more about the use of OTCs, can make mistakes without causing any harm but there are some diverse opinions on when it is the best time to incorporate MyDispense. |
| Implementation of Community Pharmacy Dispensing Software (MyDispense) in an Advanced Pharmacy Practice Cours  (Kebodeaux and Sewell, 2016) | United States | Quantitative | Survey | To study the use and application of MyDispense into a particular course, which paves the way for making future modifications in the curriculum and helps in implementing this program. | Most students appreciated the risk-free practice of MyDispense simulation, and also considered this simulation to be more realistic compared to theoretical learning. A few students believe this simulation should be incorporated throughout the curriculum. |
| The Use of Simulation to Improve IPPE-1 Performance.  (Condee and Testman, 2017) | United States | Quantitative | Survey | Primary objective: Allow students that do not have a previous experience in community pharmacy to enhance their knowledge, skills and confidence.  Secondary objective: Reduce the training burden on preceptors. | Through three lab simulations focusing mainly on the use of MyDispense, students could develop their knowledge about the top 200 medications, dispensing and counselling skills, it was found that the majority of first year pharmacy students had either little or no previous experience in pharmacy. |
| Assessing Student Performance in the Medication Use Process Using Community Pharmacy Simulation (MyDispense)  (Kebodeaux, Sewell et al, 2017) | United States | Quantitative | Observations | To study the factors which affect the students' ability in achieving competency in the basic dispensing skills. | The performance of students on assessments was high however, was negatively correlated with the total completion of MyDispense practice exercises. Additionally, student performance was not significantly impacted by the exam time. |
| A Novel Approach to Pharmacy Practice Law Instruction  (Deneff, Holle et al, 2021) | Australia | Qualitative | Survey | To study the benefits and student thoughts on MyDispense simulation of law related activities. Also, to improve the teaching process by remodeling the curriculum based on results from this study and MPJE scores of participating students. | MyDispense exercises helped students in recalling pharmacy laws and practicing areas that they found challenging. However, it was noted that exercises should have included more law concepts and scenarios. |
| A Simulated Learning Environment for Teaching Medicine Dispensing Skills  (McDowell, Styles et al, 2016) | United States | Mixed | Survey | To develop a simulation program that can mimic the dispensary so students can enhance their dispensing skills in a risk-free environment. | MyDispense enhanced students' knowledge and dispensing skills in a risk-free environment and prepared them for placements. The exercises done using MyDispense allowed students to develop their dispensing competency through the integration of cognitive and technical skills. They also developed their professional competencies required for pharmacy practice. |
| Analysis of Dispensing Errors Made by First-Year Pharmacy Students in a Virtual Dispensing Assessment  (Chuang, Grieve et al, 2021) | Australia | Mixed | Survey | To determine the errors that are most commonly made in the dispensing process among first-year pharmacy students. In addition to identifying the impact of these dispensing errors on patient outcomes if they occurred in practice. | Errors made in dispensing appropriate drug quantities, selecting the correct patient, prescriber and number of repeats, using the correct ancillary labels and writing the appropriate label directions were analyzed by referring to an assessment done through MyDispense. The most common errors were identified in writing the label directions including drug formulation and dose frequency. The severity of errors was then classified based on their expected level of harm. |
| Analysis of Student Performance Outcomes Using Virtual Dispensing Exercises  (Brown, Kebodeaux et al, 2018) | United States, Australia | Quantitative | Survey | To compare the performance between first- and second-year pharmacy students on the same exercise that is done during their OSCE. | A better performance and more accurate response was noticed among second year pharmacy students in the medication process which included patient and prescriber fact finding. Second year students asked more questions required to reach the correct outcome and spent more time on each exercise due to performing a more comprehensive fact-finding process from the prescriber and patient compared to first year students. The difference in the results is likely due to the increased exposure of second year pharmacy students to controlled prescriptions and dispensing laws. |
| Experiential learning in community pharmacy: Online and remote teaching experience in Malaysian higher education remote teaching experience in Malaysian higher education  (Rahman, Nazar et al, 2020) | Malaysia | Qualitative | Discussion | To assess student performance using a virtual community setting simulation. | The virtual community setting of MyDispense benefitted pharmacy students learning. However, this could be further improved by considering factors such as students' specific community, culture, and setting as well as how they develop their communication skills. |
| Impact of virtual simulation in self-care therapeutics course on introductory pharmacy practice experience self-care encounters  (Tai, Rida et al, 2020) | United States | Mixed | Survey | To investigate whether the use of MyDispense can impact the frequency of interactions, confidence, and student performance during second-year community pharmacy introductory pharmacy practice experiences. | Students were more confident in performing self-care interactions after using MyDispense, especially in the process of collecting and assessing data. Through the use of MyDispense, it was reported that students could develop their level of care, selection of the medication regimen, counselling, and creation of follow-up plans. |
| Implementation of a virtual dispensing simulator to support US pharmacy education  (Ferrone, Kebodeaux et al, 2017) | United States | Qualitative | Survey | To adapt its use for US schools of pharmacy and measure student perceptions in these schools. | Most students agreed that MyDispense is easy to learn, provides more realistic cases compared to paper assessments and was good for practicing without risk of patient harm. |
| Integration of a Community Pharmacy Simulation Program into a Therapeutics Course  (Shin, Tabatabai et al, 2018) | United States | Mixed | Survey | To demonstrate the effect of MyDispense on student perceptions and learning. | There was no significant difference in test scores in students who completed MyDispense and those who completed a traditional paper, though higher confidence was reported in the MyDispense group. |
| Integration of a Virtual pharmacy Dispensing Simulator ''MyDispense'' in an Experiential Education Program to Prepare Students for Community Introductory Pharmacy Practice Experience  (Johnson, Barrack et al,2021) | United States | Mixed | Survey | To evaluate the effectiveness of MyDispense in preparing students for community introductory pharmacy practice experiences. | MyDispense provided a more realistic dispensing experience and opportunity for students to make mistakes with no risks to the patient. Students also found that MyDispense helped improve their counselling skills. |
| Integration of a virtual pharmacy simulation program “MyDispense” in clinical pharmacy education  (Aksoy and Öztürk, 2021) | Turkey | Quantitative | Survey | To determine the influence of MyDispense on the clinical pharmacy educational outcomes of pharmacy students and to identify the satisfaction and confidence levels of students towards the integration of simulation into their learning. | Satisfaction' scores increased favorably post-test, as did their confidence and motivation. There was a significant difference in students' perception of clinical experience measures except for two, whilst 'decision-making and technical abilities' showed significant differences across all measures. |
| International deployment of a virtual dispensing simulator supporting pharmacy education.  (Ferrone, Brock et al, 2015) | United States | Mixed | Survey | Virtual simulation provides students with the opportunity to practise various skills and competencies in a safe environment. UCSF and UConn employed MyDispense to teach dispensing skills. | The majority of the first-year pharmacy class (98%) communicated their satisfaction of the online simulation- affording them the opportunity to make dispensing mistakes and learn from feedback. Overall, learners reported that the software was easy to use, that it enhanced their knowledge and skills of dispensing, and that it should be used early and often in a pharmacy curriculum. Preceptors also suggested that the tool raised the preparedness of students completing introductory placements. |
| Learners' Perceptions on Virtual Simulation Using MyDispense in the Philippines  (Amatong, Asentista et al, 2021) | Sri Lanka | Mixed | Survey | To determine the learners' level of perception on virtual simulation by assessing the participation, interaction, accessibility, flexibility, and adaptability using MyDispense. | The results showed that respondents were mostly female (78.9%), in their second year of college (55.90%), had internet speed of 3-5mbps (38.51%), and a College from the Southern Part of the Philippines (83.54%). High levels of perceptions on MyDispense for virtual dispensing practice were expressed as mean values of learner's participation (3.97), interaction (3.47), accessibility (3.53), flexibility (3.55), and adaptability (3.76). Moreover, significant relationships (p<0.05) were found in participation & internet connectivity (0.000); accessibility & internet Connectivity (0.05); flexibility & institution (0.000); and adaptability & year level (0.030). |
| MyDispense impact in compensating summer field training course during COVID-19 pandemic  (Al-Hindi and Mojally, 2021) | Saudi Arabia | Mixed | Survey | To study the opinion of summer training pharmacy students on the influence of replacing real field training with MyDispense. | The results showed that students needed further training to use this simulation accurately. MyDispense could not completely replace real-training. |
| MyDispense: Lessons from Global Collaboration in Developing a Pharmacy Educational Simulation Tool  (Costelloe, 2017) | Australia | Qualitative | Discussion | To build a tool which is freely accessible and has an international relevance to achieve global collaboration. | Discussed the value of global collaboration for educational initiatives like MyDispense. Monash saw value in making this free for other universities to access and enhance. No cost also made MyDispense accessible to developing countries. Academic relationships were formed from local collaboration. Suggestions for anyone creating a similar global / education project were outlined. |
| MyDispense: Taking pharmacy education into the future together  (Burton, 2021) | South Africa | Qualitative | Discussion | To look into the benefits of MyDispense in pharmacy education and skills learnt from it. In addition to the implementation of this program in South Africa and its international use, particularly during the COVID pandemic. | The software was used in 32 pharmacy schools. However, during COVID-19 where demand increased internationally, there are currently 120 pharmacy schools worldwide that are utilizing the software now. |
| Use of MyDispense Among Pharmacy Programs Across the United States  (Phanudulkitti, Kebodeaux et al, 2022) | United States | Quantitative | Survey | To describe the use of MyDispense in pharmacy education across the United States. | 50% of responses indicated the use of MyDispense in their program had increased during the COVID-19 pandemic. Most common skills: Dispensing, patient communication, drug-information skills; Most common exercises: Validation exercises, dispensing, and OTC exercises; More collaboration between instructions is recommended to develop this simulation further. |
| Virtual simulation to personalize student learning in a required pharmacy course  (Ambroziak, Ibrahim et al, 2018) | United States | Mixed | Survey | Primary objective: To incorporate virtual simulation cases which focuses on the dispensing process to patients in the Pharmacy Practice Skills I course for first year Doctor of Pharmacy students. Additionally, researchers wanted to determine if the previous pharmacy experience impacted the use of MyDispense. Secondary objectives: To determine if the previous use of virtual experience and pharmacy experience had an effect on the virtual simulation component of the final examination. Moreover, they wanted to study students' perceptions of their learning using MyDispense. | MyDispense allowed students to understand how much more practice each of them needs in order to obtain specific skills helping with medication dispensing. Results showed that both groups of students (With prior work experience/ No work experience) found the software beneficial. |
| What now and what next? The new era of OSCE  (Ali, 2020) | Saudi Arabia, Australia | Qualitative | Survey | To evaluate the alternate methods of OSCE compared with traditional face-to-face ones. | MyDispense was used to conduct web-based OSCEs in pharmacy education during the pandemic. Students determined this gave a better learning experience than face-to-face OSCEs, mainly due to the instant detailed feedback they received. Students missed the face-to-face aspect of teaching. |
| Educational Methods and Technological Innovations for Introductory Experiential Learning Given the Contact-Related Limitations Imposed by the SARS-CoV2/COVID-19 Pandemic  (Reynolds, Rhein et al, 2021) | United States | Mixed | Survey | To compare the use of experiential learning with in-person learning through the adapted curriculum. | My Dispense was shown to be helpful when integrated into a revised IPPE course (due to COVID restrictions). Students agreed that educational outcomes were met and were relevant to pharmacy practice. From the student's perspective, there was also a slight improvement in knowledge, skills and abilities. However, a higher baseline standard of pharmacy students led to a perception of minimal improvement. |
| Meeting pharmacy educational outcomes through effective use of the virtual simulation MyDispense    (Mak, Fitzgerald et al, 2021) | Australia, United States | Qualitative | Survey | To evaluate the impact of MyDispense on student performance. | The use of MyDispense enhanced lessons and complemented experiential education, and the challenges of virtual simulation for pharmacy educators. |
| Simulated learning: Integrating clinical knowledge into the dispensing process  (Klitsie, 2019) | South Africa | Qualitative | Focus groups | To explore methods MyDispense can be utilized in facilitating the integration of cognitive skills into the dispensing process. | Simulation based education is useful in integrating cognitive skills into the dispensing process, preparing students for future practice. MyDispense can help with pharmacy practice, pharmacy law and ethics, patient communication and pain management. MyDispense is also useful in helping students to develop clinical decision-making, clinical knowledge as well as applying theory into practice. Students can also receive immediate feedback and develop multiple aspects of dispensing skills. |
| Using MyDispense to simulate validation of controlled substance prescriptions in a pharmacy law course  (Mospan and Gillette, 2020) | United States | Quantitative | Survey | To study the impact of completing MyDispense exercises on the performance of students on a law examination. | Statistically significant positive relationships between student performance and the number of MyDispense exercises completed. |
| Using Technology to Enhance Teaching and Learning in Pharmacy Education  (Kebodeaux and Mak, 2021) | United States; Australia | Qualitative | Survey | To highlight the use of innovative practices within pharmacy schools, especially during the COVID-19 pandemic. | This simulation can act as a good replacement for in-person exercises during COVID-19 period. It is also correlated with better student performance as well as a good tool for students to reduce errors in practice. This is also a good tool for educators to teach pharmacy laws. |
| Effects of virtual simulation on students' ability to assess self-care patient cases  (Mazan, Komperda et al, 2018) | United States | Quantitative | Survey | To evaluate the virtual simulations impact on the ability to assess and formulate a plan for patients of pharmacy students as well as to describe their perceptions on the use of virtual simulation in pharmacy education. | There was no significant difference between students who used MyDispense and those who did not use this simulation. However, 90% students agreed or strongly agreed that they can apply into their future practice what they learned in this simulation. |
| Integration of MyDispense in a Doctor of Pharmacy curriculum in the U.S.: Lessons learned  (Komperda and Fjortoft, 2018) | United States | Qualitative | Survey | To determine the appropriateness of MyDispense in developing students' ability to process prescriptions in the community practice setting. | Pharmacy students were satisfied with the use of simulation. Further integration of this simulation into the curriculum is recommended. |
| Connecting two pieces of separate puzzles: A MyDispense experience  (Seubert, Gill et al, 2018) | Australia | Qualitative | Survey | To evaluate how quickly tutors and students get familiarized with MyDispense through training workshops and their preferences compared to the old model. | Pharmacy students preferred the use of MyDispense compared to the old model. Based on that, reevaluating tutorial learning outcomes was encouraged. |
| Integration of MyDispense in an experiential education programme to improve student preparedness of prescription processing and medication safety  (Barrack, Johnson et al, 2018) | United States | Qualitative | Survey | To evaluate students’ readiness for community introductory pharmacy practice experiences (IPPEs) after using MyDispense. | The simulation had a great impact on students' counselling skills. However, students were marked lower by preceptors compared to their own given marks. |
| Use of MyDispense pharmacy simulation programme in integrated review of pharmacy law  (Deneff, Holle et al, 2018) | United States | Mixed | Survey | To evaluate students’, need for learning pharmacy law instruction and the use of MyDispense on developing this knowledge area. | Most students agreed that MyDispense has a great impact on their understanding of pharmacy laws. However, some exercises were considered irrelevant to practice by students. |
| Implementation of a virtual dispensing system (MyDispense) into the M.Pharm. curriculum at the University of Manchester  (Worrall, Silkstone et al, 2018) | United Kingdom | Mixed | Observations | To analyze student engagement via the completion rates from United Kingdom schools of pharmacy. | High engagement was found. More than three-quarters of students completed a MyDispense activity and at least more than 50% students completed simulated activities regularly. |
| Use of online simulation in a required self-care therapeutics course  (Rida, Tai et al, 2018) | United States | Mixed | Survey | To incorporate the use of MyDispense in teaching first-year pharmacy students and to evaluate student’s ability to provide self-care recommendations through the use of this simulation. | There were mixed feelings on whether MyDispense was a valuable tool for learning the course material. Higher confidence in completing the dispensing process was found in students who had experience working in community pharmacies. |
| Use of MyDispense to dispense extemporaneously - prepared formulations  (Nicolazzo, Mak et al, 2018) | Australia | Qualitative | Observations | To develop students’ dispensing skills to prepare extemporaneous formulations using MyDispense. | The process of using MyDispense was considered seamless and developed upon Year 1 dispensing activities based on student feedback. |
| Collaborative development of a virtual Pharmacy Practice skills laboratory at the University of Zimbabwe School of Pharmacy  Monera-Penduka, Sukwe et al, 2018) | Zimbabwe | Mixed | Observations | To improve pharmacy graduates’ clinical skills and confidence at the University of Zimbabwe through the use of MyDispense. | Many drug and patient profiles were created for adaptation to the healthcare system in Harare. Students felt this simulation could prepare them for real world practice. |
| Student pharmacist performance on an Objective Structured Clinical Examination (OSCE) using community pharmacy simulation (MyDispense)  (Kebodeaux, Brown et al, 2018) | United States | Quantitative | Observations | To evaluate student performance on an Objective Structured Clinical Examination (OSCE) using MyDispense to measure competency on the medication use process. | The analysis was in progress, no results were confirmed. |
| Use of a virtual pharmacy simulation (MyDispense) for teaching dispensing skills in first-year pharmacy students  (Mak, Beaumont et al. 2018) | Australia | Quantitative | Observations | To determine first year pharmacy students' dispensing completion and accuracy rates. | There was a high rate (>95%) of correct medication/patient/dose selected by students. 63% students who scored above 95% in their dispensing assessments completed a total of 7000 exercises prior to the assessment. |
| Effects of virtual simulation on student pharmacists' ability to assess self-care patient cases (Mazan, Komperda et al, 2022) | United States | Quantitative | Observations | To evaluate the effectiveness of virtual simulation on student pharmacists' ability to assess and formulate a plan for patients seeking self-care | Students completed MyDsipense OTC exercises. Their results were compared to their colleagues from the previous year who did not use MyDispense. Results showed no difference in scores between both groups. Students in the previous year only scored differently for case 4, which was lower in the MyDispense group. However, students in the MyDispense group asked more questions about OTC, herbal and vitamin use' more so than the control group |
| An Introductory Over-The-Counter Simulation For First-Year Pharmacy Students Using A Virtual Pharmacy (Rude, Eukel et al, 2022) | United States | Mixed | survey | To assess the impact of the OTC simulation on students’ knowledge and confidence of OTC medications and overall perceptions of the activity. | Pre and post survey scores indicated that students' knowledge scores increased. Students confidence was also increased after the completion of MyDispense OTC simulation. Student perceptions were overall very positive. |
| Incorporation of MyDispense, a Virtual Pharmacy Simulation, into Extemporaneous Formulation Laboratories (Nicolazzo, Chuang et al, 2022) | Australia | Quantitative | Observation | To evaluate the outcomes of Incorporation of MyDispense, a Virtual Pharmacy Simulation, into Extemporaneous Formulation Laboratories | 100% of students came prepared to the labs with printed labels from MyDispense which provided more time for hands-on extemporaneous activity. |
| A comparison between student performances on objective structured clinical examination and virtual simulation (Amirthalingam, Hamdan et al, 2022) | Saudi Arabia | Mixed | Questionnaire | To compare the pharmacy students' performance on an interactive web-based virtual pharmacy tool versus in-person objective structured clinical examination (OSCE) | There was a significant increase in the average test scores from the virtual MyDispense exam than the in-person OSCE. |
